# Complexity–biodiversity relationships on marine urban structures: reintroducing habitat heterogeneity through eco-engineering

**DOI:** 10.1098/rstb.2021.0393

**Published:** 2022-08-15

**Authors:** Melanie J. Bishop, Maria L. Vozzo, Mariana Mayer-Pinto, Katherine A. Dafforn

**Affiliations:** ^1^ School of Natural Sciences, Macquarie University, North Ryde, NSW 2109, Australia; ^2^ Sydney Institute of Marine Science, Mosman, NSW 2088, Australia; ^3^ Centre for Marine Science and Innovation, School of Biological, Earth and Environmental Sciences, University of New South Wales, Kensington, NSW 2052, Australia

**Keywords:** habitat complexity, eco-engineering, environmental gradient, microclimate, microhabitat, urbanization

## Abstract

Urbanization is leading to biodiversity loss through habitat homogenization. The smooth, featureless surfaces of many marine urban structures support ecological communities, often of lower biodiversity, distinct from the complex natural habitats they replace. Eco-engineering (design for ecological co-benefits) seeks to enhance biodiversity and ecological functions on urban structures. We assessed the benefits to biodiversity of retrofitting four types of complex habitat panels to an intertidal seawall at patch (versus flat control panels) and site (versus unmodified control seawalls and reference rocky shores) scales. Two years after installation, patch-scale effects of complex panels on biodiversity ranged from neutral to positive, depending on the protective features they provided, though all but one design (honeycomb) supported unique species. Water-retaining features (rockpools) and crevices, which provided moisture retention and cooling, increased biodiversity and supported algae and invertebrates otherwise absent. At the site scale, biodiversity benefits ranged from neutral at the high- and mid-intertidal to positive at the low-intertidal elevation. The results highlight the importance of matching eco-engineering interventions to the niche of target species, and environmental conditions. While species richness was greatest on rockpool and crevice panels, the unique species supported by other panel designs highlights that to maximize biodiversity, habitat heterogeneity is essential.

This article is part of the theme issue ‘Ecological complexity and the biosphere: the next 30 years’.

## 1. Introduction

Habitat complexity (hereafter ‘complexity')—the number of different structural elements per unit area—is widely regarded as a key driver of community composition and a positive determinant of biodiversity [1–3]. Complexity, through the creation of microhabitats, increases the range of niches available to species [4], and hence the number of species that can recruit into an area [5]. In stressful environments, complexity can increase organismal survival by providing protective structure that mitigates predation and/or key environmental stressors [3,6–8]. Additionally, complex habitats often (though not always) have a greater surface area for organismal attachment and grazing, thereby increasing resource availability and reducing the effects of competition [3].

In the sea, as on land, urbanization is simplifying habitats and reducing their complexity [9,10]. This simplification is in part caused by the destruction and degradation of complex natural habitats, such as mangrove forests, seagrass beds, and coral and shellfish reefs, and their replacement with built infrastructure [11]. Structures such as seawalls, revetments, pilings and pontoons are built in growing numbers to protect coastal settlements from rising sea-levels, to support the booming blue economy, and as part of land reclamation efforts to accommodate growing coastal populations [12]. In urbanized coastal environments, seawalls and other forms of shoreline hardening now armour over 50% of the shoreline [13,14]. Urban structures differ from natural habitats in the materials from which they are constructed but are also typically characterized by smooth, featureless surfaces [15]. At scales of millimetres to tens of metres, seawalls have lower structural complexity than natural rocky shores, and at the smallest and largest scales, rock revetments are similarly deficient [16].

The low complexity of marine urban structures has the potential to drive marked and pervasive changes in marine benthic communities [15]. Biofilms of diatoms and cyanobacteria, as well as invertebrates and macroalgae, settle, feed and grow on hard substrates [15]. These provide food and habitat to mobile invertebrates and fish [8,17]. As compared with natural hard substrates, urban structures typically support distinct ecological communities, often of reduced native biodiversity, enhanced non-native biodiversity and fewer ecological functions [15,18,19]. Consequently, there has been growing interest and investment in enhancing the topographic complexity of coastal defence structures, such as seawalls, to enhance biodiversity and the desired ecosystem services it provides [6,20–23].

Enhancement of the complexity of marine urban structures is ideally done at the time of structure design [24], but for the tens of thousands of kilometres of coastline that is already hardened it can also be ‘eco-engineered' (designed for ecological co-benefits) through retrofits of habitat-enhancing units or modification of the existing structure's surface [6,25,26]. To date, enhancement of complexity through eco-engineering has predominantly been applied at small, experimental scales, to existing structures (but see [27] for a larger-scale example) and limited to adding a single type of microhabitat (e.g. water-retaining feature, pits, holes [6], but see [8,23] for examples simultaneously manipulating two types of complexity). Despite the positive effects of complexity that have been described at seascape and landscape scales [3], these interventions have produced effects on biodiversity ranging from highly positive to neutral [6,21,28] and on individual functional groups of organisms that range from positive to negative [21].

Whether complexity facilitates or inhibits a particular group of species will be dependent on the match between the species' niche and the microhabitats provided [29]. For example, whereas the incorporation of cool and dark water-retaining features such as rock pools into a structure may benefit invertebrates that are sensitive to desiccation stress, it may negatively impact those species of algae that require a threshold amount of light for photosynthesis [21]. Similarly, whereas small interstices produced by rock weathering patterns, may offer species of small body size protection from larger-bodied predators, they will not protect species whose body size exceeds the size of the interstices, or those that are targeted by small-bodied prey [7]. Consequently, at the patch scale, the community supported by a habitat may vary according to the type of complexity provided [30], and at the site scale, biodiversity is predicted to be greater where multiple types of complexity are provided together (i.e. habitat heterogeneity is high) than when a single type of complexity is provided alone (i.e. habitat heterogeneity is low).

Additionally, ecological benefits of complexity may depend on its efficacy in mitigating key environmental stressors [7,21]. On intertidal rocky shorelines, biodiversity is broadly regarded as being shaped by the paradigmatic gradients of abiotic (desiccation and thermal) stress, which increases from the low to the high shore, and biotic stress (competition and predation by marine species), which often runs counter to this ([31,32], but see [33,34]). For the many rocky intertidal species living at or close to their upper thermal limit [35], survival can be dependent on the availability of thermally buffered microhabitats [36]. In environments with strong top-down control, species must either rely on anti-predator defences (e.g. thick shells; [37]), or reside in protective microhabitats [38]. Consequently, types of complexity that are effective at providing cool, moist microclimates, or that exclude finfish or shorebird predators, may be particularly effective at increasing intertidal biodiversity.

Here, we assess how retrofitting complex habitat to seawalls influences biodiversity at the patch and site scales. Specifically, we attached complex habitat panels of four different designs and flat control panels across the tidal elevation gradient of a seawall in Sydney Harbour (hereafter referred to as the eco-engineered seawall). We then compared ecological communities at the eco-engineered seawall and at unmodified (control) seawalls and natural (reference) rocky shores. We hypothesized that: (i) over time complex panels would acquire a greater number of species (i.e. species richness) and abundance of mobile and sessile invertebrates and algae than flat (control) panels; (ii) complex panels of differing designs would support communities distinct from one another; (iii) differences in community structure among panel designs would be driven by the availability of microhabitats that mitigate heat and desiccation stress; and (iv) at the site scale, eco-engineered seawalls would develop greater species richness and distinct assemblages compared with unmodified control seawalls, and similar richness and assemblages to reference rocky shores.

## 2. Material and methods

### (a) Study sites

**Figure 1.**
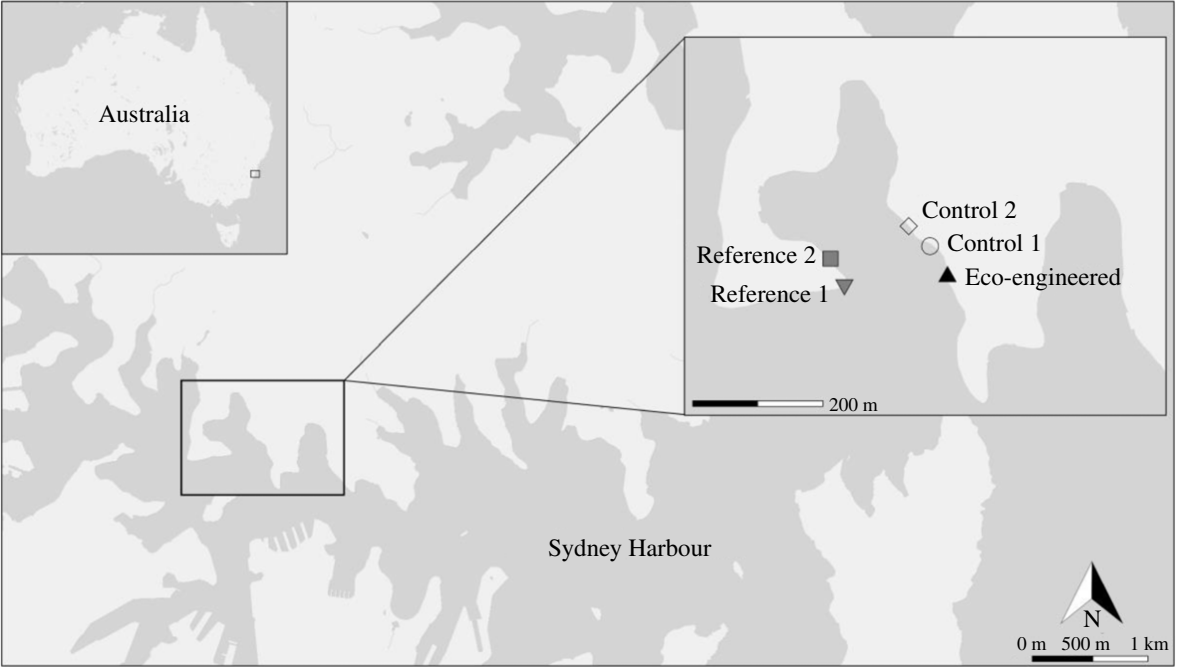
Location of study sites within Sydney Harbour, New South Wales, Australia.

The study was conducted at five sites on the northern side of Sydney Harbour, New South Wales, Australia: one vertical seawall site installed with complex panels (hereafter ‘Eco-engineered site'), two otherwise similar vertical seawall sites unmodified by panels (hereafter ‘Control sites') and two reference rocky shore sites with a mixture of vertical and sloping horizontal surfaces (hereafter ‘Reference sites'). The Eco-engineered and Control sites were located at Sawmillers Reserve (33.845702S, 151.201062E), and the Reference sites at Balls Head Reserve (33.846760S, 151.197988E) (figure 1). The sites were situated 5–8 km from the mouth of the estuary and had a tidal range of approximately 1.8 m. Each site was 12 m in length and separated from other sites by at least 50 m and by no more than 1 km.

**Figure 2.**
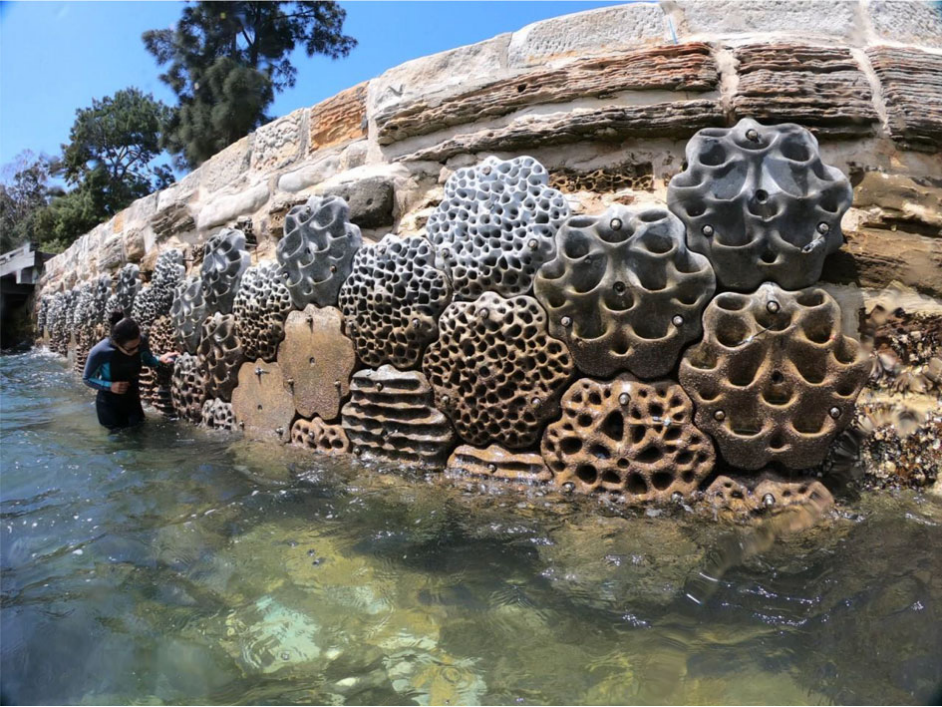
The Eco-engineered site, approximately 6 months following panel installation (photo credit: Alex Goad). (Online version in colour.)

**Figure 3.**
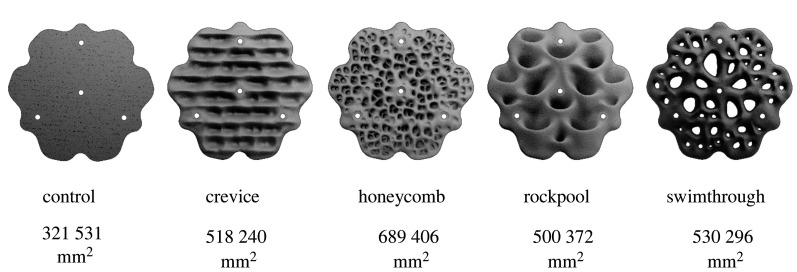
The five panel designs used in experiments and the area of their front (outer-most) surface. All panels were 550 mm in diameter, had flat backs and had the same projected area of 321 531 mm^2^.

The Eco-engineered and two Control sites were situated on the seawall at Sawmillers Reserve. This vertical intertidal seawall was approximately 320 m long and had approximately 2 m of intertidal zone. At the Eco-engineered site, seventy-two 550 × 520 × 100 mm concrete ‘Living Seawalls' panels designed by Reef Design Lab (Melbourne, Australia) were installed in November 2018 along a 12 m length stretch of shoreline (figure 2). Panels of five different designs were fitted in a continuous mosaic that covered the entire intertidal zone (approx. 2 m height; electronic supplementary material, figure S1). These included four complex designs as well as a control (flat panel, free of any macro-scale habitat complexity) (figure 3). The complex designs had microhabitat features that mimicked those of intertidal rocky shores, the closest natural analogue of seawalls, specifically: (i) crevices and ridges (horizontal depressions separated by ridges), (ii) honeycomb (less than *ca* 25 mm pitting across the panel surface), (iii) rockpools (15 cup-like structures per panel designed to hold water as the tide retreats), and (iv) swimthrough (multiple different-sized holes allowing organisms to pass through the panel). Panels were attached to seawalls using three stainless steel rods drilled into the seawall such that the panels sat approximately 10 cm off the surface of the wall, eliminating the need to clear existing marine life on the seawall or level the seawall prior to panel installation.

### (b) Panel-scale assessments

Panel-scale sampling tested the hypotheses that within the Eco-engineered site: (i) complex panels would support greater richnesses and abundances of species than flat, control panels and that these differences would strengthen through time as more species colonized; (ii) complex panels of differing designs would support distinct benthic communities; and (iii) differences in communities among complex panels would reflect differences in the microhabitats present, and not be explained by differences in surface area alone. Four replicate panels of each of the five designs (control, crevices, honeycomb, rockpool and swimthrough (figure 3)) were repeatedly sampled at the mid-intertidal elevation (mean low water springs (MLWS) + 0.5 m) of Sawmillers Reserve at 2, 4, 6, 8, 12 and 24 months following panel deployment. Low- and high-intertidal elevations were not considered in panel-scale assessments as not all designs were represented within these.

First, to test for differences in communities among panel designs, four panels of each design were sampled *in situ* using a 25 × 25 cm quadrat with 25 intersecting points, which was placed over their centre. Within each quadrat, the per cent cover of sessile biota was assessed using the point-intercept method and mobile biota were counted. To represent the full sessile community, both primary (i.e. attached directly to the substrate, i.e. panel) and secondary (i.e. growing on the primary species) cover were recorded separately and summed to give a total cover that could exceed 100%. Sessile biota that were present within the quadrat but did not fall beneath a point intercept were given a count value of 0.1 (0.4%) to note their presence. All organisms were identified to species, or where this was not possible, morphospecies.

Second, to test whether differences in sessile and mobile communities among complex panel designs reflected differences in microhabitats, we subsampled inner (e.g. inside crevices, rockpools, depressions or holes) and outer surfaces (ridges, and outside of rockpools, holes and depressions) of complex panels. Microhabitats were subsampled on four replicate panels of each of the complex designs (i.e. crevice, honeycomb, rockpool and swimthrough) at 2, 4, 6, 8, 12 and 24 months following panel installation. For the first five time points (i.e. until 12 months) six replicate mini-quadrats were haphazardly sampled within each of the two microhabitats per panel. At 24 months, this was reduced to three replicates per microhabitat per panel, owing to the substantial growth that slowed sampling. Microhabitats were sampled using 4 × 4 cm quadrats, constructed of transparent film that was marked with 3 × 3 evenly spaced lines to create nine intersection points. The quadrats could be moulded to fit the contour of microhabitats. Sessile and mobile species were quantified within quadrats using the methods described above. For each time point, we averaged mini-quadrats across each microhabitat of a panel to obtain a single estimate of community structure for the inside versus outside microhabitat of each panel.

To test for differences in the thermal environment between the inner and outer microhabitats of each complex panel design and the surface of the flat panel, a single iButton data logger (DS1921G, Thermodata, Warrnambool Australia) was randomly positioned in each microhabitat of each of three replicate panels per design, at the mid-intertidal elevation. The loggers, waterproofed with Plastidip rubber coating (Plasti Dip International, Blaine, Minnesota, USA), were programmed to record temperatures with 0.5°C accuracy at 20 min intervals during December 2018–February 2019 and increased to 60 min intervals from February 2019. Loggers were attached to panels using Fisher FIS EM Plus 390 S epoxy mortar and were interchanged approximately every 4–10 weeks to provide near-continuous monitoring of temperature throughout the experiment.

### (c) Site-scale surveys

Site-scale sampling was conducted to test the hypotheses that: (i) prior to panel installation, the Control and Eco-engineered sites would support fouling communities distinct from those at Reference sites, and (ii) after panel installation, the Eco-engineered site would initially support communities distinct from those at either Control or Reference sites, but through time acquire communities more similar to those at the Reference sites. To test these hypotheses, each of the five sites (2 × Control; 2 × Reference; 1 × Eco-engineered) was sampled twice before (6 and 1 month prior) and four times after (6, 12, 18 and 24 months) installation of habitat panels. The exception was Control Site 2 and Reference Site 2, which were only sampled from 6 months onwards (i.e. the four ‘after' times). At the Eco-engineered site, the sampling was done on the unmodified seawall before and on the panels after installation.

Owing to strong vertical gradients in communities, sampling of sessile and mobile communities was stratified by tidal elevation. Three intertidal elevations were sampled at each site: high (MLWS + 0.9 m); mid (MLWS + 0.6 m); and low (MLWS + 0.3 m). At the two before-installation times, five replicate 25 × 25 cm quadrats were haphazardly sampled per elevation of each site, at low tide. For the four sampling times after installation of panels, this was increased to 10 replicate quadrats to better document biodiversity. Quantification of sessile and mobile species within quadrats was as described for the panel-scale sampling.

### (d) Statistical analysis

Multivariate and univariate permutational analyses of variance (PERMANOVA) [39] tested hypotheses about panel- and site-scale effects of complex panels on biodiversity. Multivariate analyses used Bray–Curtis dissimilarity matrices that were produced on untransformed matrices of each of: (i) sessile species percentage covers and (ii) mobile species abundances. Univariate analyses, using Euclidean distances, were run on each of: (i) species richness (for sessile and mobile species combined, and independently); (ii) the total cover of fouling species; (iii) the total abundance of mobile species, and for panel-scale analyses; and (iv) the covers of key functional groups of habitat-forming species identified in our study that were abundant enough to analyse (red non-calcified algae, coralline algae, brown algae, green algae, barnacles, oysters and tube worms).

The development through time of panel-scale effects of complexity on mid-intertidal communities was assessed using three-way analyses, with the factors: Panel design (fixed); Time (fixed) and Panel identity (random, nested in Panel design). Panel identity was included as a factor in analyses as the same panels were repeatedly sampled through time. Time was considered a fixed factor as we hypothesized that differences among panels would strengthen through time as additional colonization occurred. These analyses were run on all multivariate and univariate metrics, with the exception of functional group covers, which were instead run separately on 12 and 24 month data and included only the factor Panel design. This approach was taken because of the low abundances of some taxa at earlier sampling times.

To assess the extent to which panel-scale differences were driven by differences in protective microhabitats, we conducted a second set of analyses that compared the inner and outer microhabitats of complex panel designs (as sampled using mini-quadrats). These analyses had four factors: Panel design (fixed; four levels, excluding controls that were not sampled), Microhabitat (fixed; inner versus outer), Time (fixed) and Panel identity (random, nested in Panel design). Similar analyses, but also including the Control microhabitat, were run on the mean, maximum and minimum temperatures recorded by loggers each calendar month. Analyses of temperature variables excluded the factor Panel identity, as loggers were rotated among panels and time points were hence temporally independent.

Finally, site-scale effects of complexity were assessed using three-way permutational analyses of variance (PERMANOVAs) with the factors Treatment (Eco-engineered, Control, Reference), Site (nested within Treatment) and Time (fixed). Before versus After was not included as a factor in our analysis owing to predictions of temporal change at the Eco-engineered site following panel installation as well as lack of replication of the Control and Reference sites prior to panel installation (i.e. only one Control and one Reference site were sampled before). A separate analysis was run on data from each of the three tidal elevations, as differences in communities among these otherwise dominated analyses.

For all analyses, sources of significant differences were assessed using *a posteriori* pairwise PERMANOVAs. In instances where too few permutations (i.e. fewer than 100) were available for a given test, *p*-values were obtained through Monte Carlo simulations [40]. Similarity percentages (SIMPER) analyses in PRIMER [39] were used to identify key discriminating taxa (dissimilarity to standard deviation ratio greater than 1.3) driving multivariate treatment effects. All analyses were run in PRIMER v7 with PERMANOVA+.

## 3. Results

### (a) Panel-scale effects of complexity

**Figure 4.**
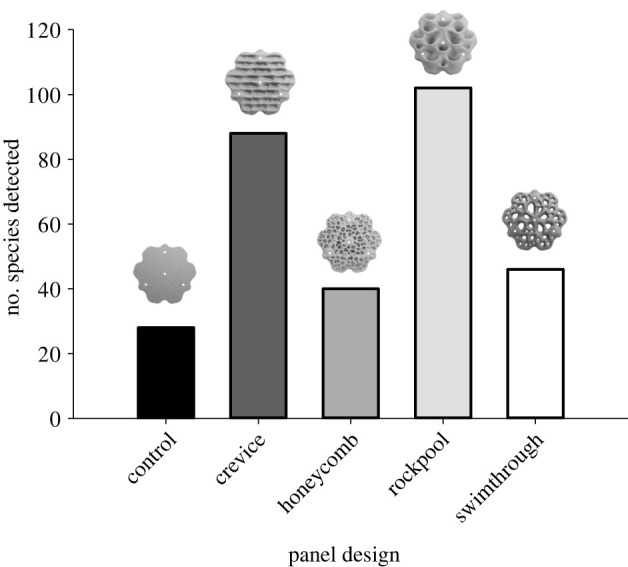
The total number of species detected on each of the five panel designs, at a mid-intertidal elevation, over the 24 month study.

At the panel scale, we found a total of 115 species over the 24 month study, including 25 species of algae, 42 species of sessile invertebrate and 48 species of mobile invertebrate (electronic supplementary material, table S1). Of these, 33 species were unique to a single panel design, with rockpool panels supporting 22 of these, crevice panels 9 and swimthrough panels 2 unique species (electronic supplementary material, table S1). Among the five panel designs, rockpool panels supported the greatest number of species (102 detected across all panels during the experiment) and control panels the least (28; figure 4).

**Figure 5.**
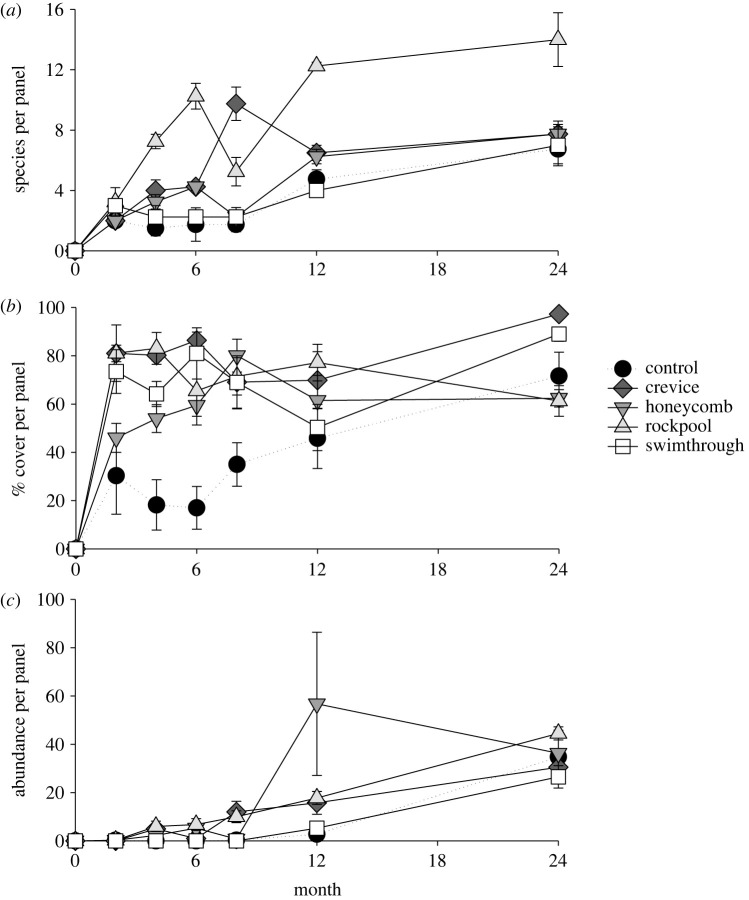
Mean (±1 s.e.) (*a*) number of taxa, (*b*) per cent cover of sessile species, and (*c*) count of mobile species per quadrat, 0–24 months following panel installation. *n* = 4.

Analyses of species richness per panel revealed that the pattern of greatest richness on rockpool panels was apparent from 4 months post-installation and persisted to 24 months (significant Panel design × Time interaction, pseudo-*F*_70,23_ = 7.0, *p* = 0.001; figure 5*a*). Although the crevice and honeycomb panels also initially (at 4 months) had higher species richness than control panels, honeycomb panels were statistically indistinguishable from control panels by 6 months, and crevice panels by 12 months (electronic supplementary material, table S2; figure 5*a*). Initially, species richness on each of the panel designs increased with time since installation, but between 12 and 24 months species richness stabilized (electronic supplementary material, table S2; figure 5*a*).

Effects of panel design varied between sessile and mobile communities (electronic supplementary material, table S2 and figure S2; figure 5*b,c*). Sessile taxa initially (2–8 months post-panel installation) differed in multivariate community composition between control and complex panels, with the four complex panels (i.e. crevice, honeycomb, rockpool, swimthough) displaying few differences (electronic supplementary material, table S2 and figure S2). Across longer time-frames of 12 and 24 months, multivariate sessile communities of the honeycomb and swimthrough panels converged with the controls, though crevice and rockpool panels maintained distinct sessile communities—a pattern that persisted to 24 months (electronic supplementary material, table S2 and figure S2). Greater abundances of the algae *Ulva australis* and *Ralfsia* sp. as well as the barnacles *Austrominius modestus* and *Austrominius covertus* on rockpool and crevice than other panels (SIMPER) drove these differences.

Multivariate analyses revealed that effects of complex panels on community structure were weaker for mobile than sessile communities. Rockpool panels were the only design to consistently support mobile species communities distinct from the control panels—a pattern that was apparent from 4 months (electronic supplementary material, table S2 and figure S2). SIMPER analysis indicated that this multivariate pattern was driven by the limpets *Patelloida mimula*, *Patelloida mufria* and *Siphonaria denticulata*, each of which had greater abundances on rockpool panels, and the gastropod *Affrolittorina acutispira*, which was least abundant on the rockpool panels. By contrast, swimthrough panels did not support mobile communities distinct from control panels at any of the sampling times (electronic supplementary material, table S2 and figure S2). Crevice and honeycomb panels supported distinct communities at some intermediate time points (between 4 and 12 months), but had converged on controls by 24 months (electronic supplementary material, table S2 and figure S2; figure 5*c*).

Similar to the multivariate community structure, sessile species richness and cover were initially (until 6 months) greater on most complex panel designs than on flat controls (electronic supplementary material, table S2; figure 5*b*). The exception was sessile richness on the honeycomb panel, which was statistically indistinguishable from that on the flat control at all sampling times. However, by 24 months only the crevice and rockpool panels supported greater covers than the controls and only the rockpool panel supported greater sessile species richness (electronic supplementary material, table S2). Mirroring multivariate patterns in mobile communities, the rockpool panel was the only design to support consistently higher abundances of mobile gastropods than did control panels, though crevice panels supported intermediate abundances to control and rockpool panels at 4–12 months (electronic supplementary material, table S2; figure 5*c*). Differences among panel designs in mobile species richness were temporally variable, and at 24 months no significant differences were seen (electronic supplementary material, table S2).

**Figure 6.**
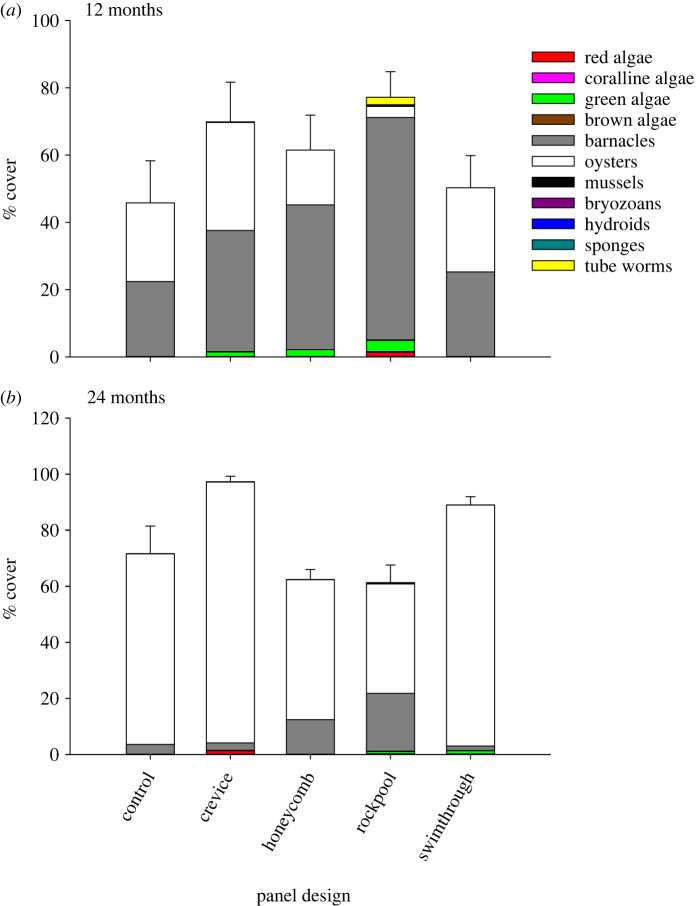
Mean (±1 s.e.) total per cent cover and cover by functional group of species on panels (*a*) 12 months and (*b*) 24 months after deployment. *n* = 4. (Online version in colour.)

At 12 months, sessile cover on each of the panel types was dominated by barnacles (mean ± s.e. cover: 39 ± 5%), with oysters the second most dominant group (20 ± 4%; figure 6). Neither group, however, significantly differed in cover among panel types (electronic supplementary material, table S3). Coralline algae, by contrast, were only found on rockpool panels, and tube worms on crevice and rockpool panels, with greatest cover in rockpools (electronic supplementary material, table S3). By 24 months, oysters were the spatial dominant on all panel types (mean ± s.e. cover: 67 ± 5%), with significantly greater abundances on crevice and swimthrough panels than the other designs (electronic supplementary material, table S3; figure 6). Barnacles, though reduced in cover on all panel types (mean ± s.e. cover: 8 ± 2%), had significantly greater cover on rockpool than other panels (electronic supplementary material, table S3; figure 6). Other groups did not display significant differences among panel designs.

At all sampling times, the inner microhabitat of rockpool panels supported a greater species richness than any of the other microhabitats (electronic supplementary material, figure S3 and table S4)—a pattern that was also apparent when sessile and mobile richness were analysed separately. No other differences in richness were apparent among the other inner or outer microhabitats, except for the crevice panels at 24 months, where richness was greater in the inside microhabitat. Mobile species counts followed a similar pattern to total species richness, being most abundant inside rockpools (electronic supplementary material, figure S3 and table S4). By contrast, sessile cover was greater on the inner than the outer surfaces of each of the panel designs, though the crevice panel supported greater cover on its outer surface than the other panel designs.

Mean temperatures were on average 0.5°C cooler in inner than outer microhabitats, and were, on-the-whole, warmest on control and coolest on rockpool panels (electronic supplementary material, figures S4 and S5). The inner surfaces of rockpool and swimthrough panels displayed thermal maxima that were up to 10°C cooler than on control panels. The control panels experienced greater temperature maxima than either the inner or outer surfaces of the complex panels, and the inner surfaces experienced less extreme maxima than the outer surfaces of complex panels (electronic supplementary material, figure S4 and table S5). Minimum temperatures varied less among panel designs and microhabitats, but were ameliorated (warmer) in the inner microhabitats of the rockpool and swimthrough panels (electronic supplementary material, figure S4 and table S5).

### (b) Site-scale effects of complexity

**Figure 7.**
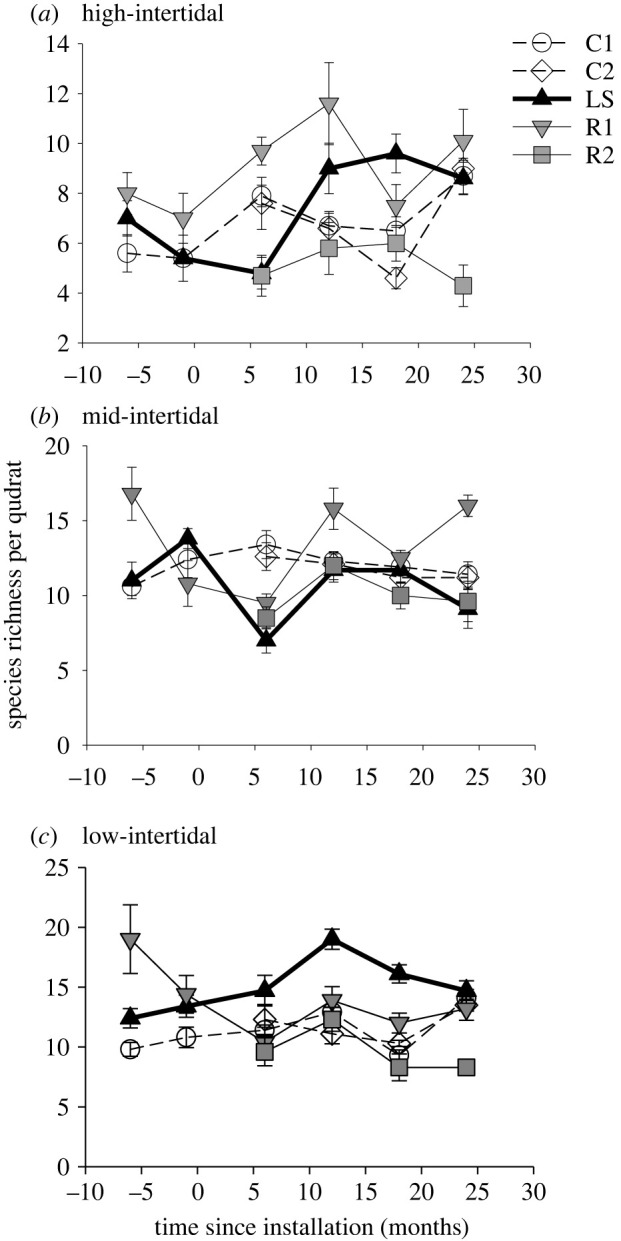
Mean (±1 s.e.) species richness per quadrat at (*a*) high-, (*b*) mid- and (*c*) low-intertidal elevations of Control (C1, C2; white), Eco-engineered (LS; black) and Reference (R1, R2; grey) sites, before (−6, −1 months) and after (6, 12, 18, 24 months) panel installation at the Eco-engineered site. *n* = 5–10.

Over the duration of the study, site-scale sampling detected 102 species of algae and invertebrates on the Eco-engineered seawall—a number that approached the most biodiverse of the two Reference rocky shores (105 species) and was greater than for either of the two Control seawalls (95, 87 species) or the second Reference rocky shore (83; electronic supplementary material, table S6). Contrary to our expectation, Reference sites did not consistently support the highest species richness of algae and invertebrates, covers of mobile species, or counts of mobile species (electronic supplementary material, table S7; figure 7). Instead, site-specific differences were detected. At the Eco-engineered seawall, we detected six species not found at any of the other sites. These included the alga *Champia* sp., the barnacle *Catmomerus polymerus*, two bryozoans, *Beania* sp. and *Cryptosula pallasinia*, the ascidian *Styela plicata,* as well as a tube worm, *Salmacina australis* (electronic supplementary material, table S6).

As expected, at mid- and low-intertidal elevations, sessile and mobile communities displayed differences in community structure between control and reference treatments (electronic supplementary material, table S7 and figures S5 and S6). By contrast, no difference in community structure was apparent among these treatments at the high-intertidal elevation. At none of the three intertidal elevations did the community of sessile species at the Eco-engineered site display change through time that was consistent with a response to the eco-engineering intervention (electronic supplementary material, table S7 and figure S5). Mobile species, similarly, displayed no community-level response to eco-engineering at high- or mid-intertidal elevations, but at the low elevation, displayed change from before to after panel installation at the Eco-engineering site that was not replicated at Reference or Control sites (electronic supplementary material, table S7 and figure S6).

Neither total species richness nor mobile species richness responded to the eco-engineering intervention at any of the three tidal elevations sampled. In the high intertidal, there was, however, a weak but non-significant trend for increase in total species richness from before to after the intervention at the Eco-engineered site, which contrasted with idiosyncratic changes at the Control and Reference sites (electronic supplementary material, table S7; figure 7*b*). By contrast, sessile species richness displayed significant responses to eco-engineering at the mid- and low-intertidal elevations.

In the mid intertidal, there was little change in species richness through time, for either of the control or reference treatments (electronic supplementary material, table S7; figure 7*b*). Richness at the Eco-engineered site initially declined immediately following installation of bare panels, but rapidly recovered to match the pre-installation value (electronic supplementary material, table S7; figure 7*b*).

In the low intertidal, sessile species richness at the Eco-engineered site gradually increased following panel installation to a peak at 12 months post-installation (electronic supplementary material, table S7; figure 7*c*). Neither of the other treatments displayed this temporal trend, with the reference treatment, to the contrary, displaying a steep decrease in richness in the time period spanning before to after installation. Consequently 6, 12 and 18 months post-panel installation the Eco-engineered site had higher richness than the other two treatments (figure 7*c*).

Eco-engineering had no effect on sessile cover or mobile abundance that could be detected above natural spatio-temporal variation (electronic supplementary material, figure S7, table S7). Indeed, the greatest change in mobile abundance at the Eco-engineered site occurred between times 6 months and 1 month prior to panel installation, so could not be attributed to eco-engineering (electronic supplementary material, table S7 and figure S7).

## 4. Discussion

Based on ecological theory [1,3] and the results of small experimental-scale marine eco-engineering studies (e.g. [8,21]), we expected that the addition of complex habitat panels to otherwise relatively flat and featureless seawalls would enhance biodiversity at panel and site scales. General effects of complexity were, to the contrary, not found by this study. Instead, effects on community assembly, richness and abundance (cover, counts) were confined to specific panel designs and tidal elevations, and exhibited considerable temporal variability. The designs offering most protection from desiccation, heat stress and predation (i.e. rockpools and, to a lesser extent, crevices), had greater richness and abundance (as compared with flat control panels) that extended for at least two years. While other designs accelerated community development and supported some unique species at some time points, their communities had converged with flat control panels by 2 years. Because all five panel designs supported unique species for at least some points in time, site-scale effects of eco-engineering on community assembly were, nevertheless, apparent. These were, however, limited to the low-intertidal elevation. These results highlight the need to match eco-engineering interventions to the niches of species and to environmental gradients in key stressors.

### (a) Panel-scale effects of habitat complexity

Complexity may enhance species richness by enhancing surface area [41], and by increasing the diversity and availability of microhabitats and hence the range of species niches supported [4–7]. In this study, whether habitat complexity enhanced species richness and appreciably altered species assembly varied through time, and according to the type of complexity provided. Initially, all complex designs, with the exception of swimthrough panels, enhanced the abundance (cover, counts) of organisms, and had more species than flat, control panels. By 24 months after panel deployment, however, only the rockpool and crevice panels supported distinct communities. The lack of a general effect of complexity suggests that its ecological effects were not driven by the enhancement of substrate surface area but instead reflected protective functions of particular microhabitats. Further, the rockpool panel, which consistently supported the greatest abundances and richnesses of species, had the second smallest surface area of the five panels.

Of the four types of complex panel, the rockpool panel was the only one designed to retain water at low tide, consequently supporting algal species and associated crustacea (amphipod, isopod grazers) that were not otherwise present. Desiccation stress is broadly considered to be among the key environmental stressors that control the upper vertical limit of species on intertidal rocky shorelines [32]. Consequently, water-retaining devices can be particularly effective at facilitating algal and invertebrate species on marine urban structures [6]. Additionally, we found that the insides of rockpools provided microclimates that were up to 10°C cooler in temperature than exposed surfaces. Crevices did not produce this same temperature-ameliorating effect, and instead their main protective function may have arisen from exclusion of key predators such as finfish, which exhibit strong top-down control on sessile invertebrates in Sydney Harbour [7,8].

The variable effects of the four complex panel designs on community structure highlight the importance of matching the ecological niche requirements of target species to the features of the microhabitats provided [42,43]. This requires identifying the key stressors to species, under a given set of environmental conditions, and identifying those aspects of complexity that successfully mitigate these [6,21]. Adding random habitat complexity to build structures, without knowledge of key environmental stressors to target species, is likely to lead to suboptimal outcomes [21,28]. For example, if the goal of an intervention is to improve water quality, crevice and swimthrough panels that have protective surfaces that bolster the cover of oysters might be selected. By contrast, if the goal is to encourage algal species, water-retaining rockpools should be selected. Because all five panel designs supported unique species for at least some points in time, biodiversity might be maximized by providing a range of types of complexity at one site. The common approach of only applying a single type of complexity to a site (reviewed by [6]) therefore represents a suboptimal approach to maximizing biodiversity.

Convergence through time of ecological communities on the various panel designs likely reflected the growing importance of competitive processes. Whereas initially communities were dominated by *r*-selected species, of high reproductive output, short life-history and broad environmental tolerances (e.g. ephemeral green algae), through time these were replaced with competitively superior *K*-selected species, such as the Sydney rock oyster, *Saccostrea glomerata* [44]. At 12 months this species occupied 20% of space, and by 24 months accounted for, on average, 67% of cover. Oysters form complex three-dimensional habitat created through the settlement of successive generations of oysters on top of one another. Consequently, as time progressed, the underlying complexity of the panels themselves was replaced by the complex structure of oysters. This complex habitat, in turn, facilitated dense and diverse communities of algae and invertebrates.

This study employed non-destructive, *in situ* sampling, as the eco-engineering intervention was designed to be permanent. Care was taken to thoroughly scan microhabitats, and our *in situ* sampling captured much of the epifaunal biodiversity associated with oyster habitat (e.g. *Patelloidia mimula* and *Bembicum auratum*, which graze on biofilms on oyster shells; and *Tenguella marginalba* and *Bedeva paivae*, which are oyster drills). Nevertheless, it is likely that both the biodiversity associated with habitat-forming species, such as oysters, and the biodiversity associated with topographically complex habitats were underestimated, resulting in conservative estimates of community differences between complex and flat panels. Destructive sampling of microhabitats and habitat-forming species would be required to fully document cryptic biodiversity, such as the infauna living in oyster biodeposits between oyster shells.

Furthermore, it is likely that ecological benefits of complex panels were underestimated by the focus of this study on fouling communities at low tide. Intertidal eco-engineering interventions can also benefit fish and mobile consumers by increasing the prey resource base, and by providing hiding places at high tide [8,45,46]. Interstitial spaces (e.g. holes), such as those provided by the swimthrough panels, may in particular be beneficial to fish biomass, abundance and species richness [43]. Indeed, the swimthrough panels were designed with fish, as opposed to fouling species, in mind.

With the exception of the swimthrough design, habitat complexity accelerated the rate of community establishment. While, ultimately, complex and flat panels may converge on similar community compositions, the acceleration of community development may assist, through the pre-emption of space, in limiting the colonization of non-native species [23,47]. Future studies would be required to test this hypothesis. Acceleration of community establishment also means that services provided by biodiversity (e.g. water filtration by bivalves, provision of food and habitat to fish) will be more rapidly recovered, and the recovery debt lowered [48].

### (b) Site-scale effects of habitat complexity

Effects of habitat complexity vary across environmental gradients, at a range of spatial scales [21,49]. Here panel-scale benefits of habitat complexity on biodiversity translated to site-scale benefits that varied spatially across a tidal elevation gradient. Positive effects of complexity on mobile communities and sessile species richness were seen on the low shore, whereas effects were weak or neutral at higher elevations. These effects were apparent within 24 months of installation despite the initially bare concrete surface of the panels, which contrasted with the many years of marine growth on unmodified seawalls and on rocky shore. Like others (e.g. [50]), we found mobile species to be particularly negatively impacted at Control sites, possibly reflecting decreased recruitment and increased mortality on urban structures [51].

Three main hypotheses may potentially explain the varying effects of complexity across the intertidal gradient. First, the greater inundation time and less abiotically stressful conditions on the low shore [31,32] may result in a more speciose pool of available colonists on which complexity can act. On the high shore, stressful conditions may, to the contrary, over-ride the effects of complexity by creating conditions that are inhibitory to the survival of most species. Other types of stressors, such as contaminants and propagule supply, have been found to mediate complexity effects [52,53]. Second, effects of predation may be greater on the low than the high shore, owing to the greater inundation time enhancing predation risk from finfish predators [54]. Third, the differing configurations of panels on the low, mid and high shore of our study site may produce differential effects. Interestingly, however, the rockpool panel, which most positively influenced species richness and abundance on the mid shore, was over-represented at the high, but not the low shore of this site. Irrespective, the stronger effects of complexity at low- than mid- or high-intertidal elevations add to growing evidence that benefits of marine eco-engineering are highly context-dependent [21,28,55].

Like panel-scale benefits of complexity, site-scale benefits peaked at 12–18 months after panel installation but were still apparent, though weaker, at 24 months. This result highlights the need for long-term studies monitoring the efficacy of eco-engineering interventions. Most studies evaluating effects of marine eco-engineering are terminated at 12 months [6,7]. Such short-term studies may over-inflate the benefits of marine eco-engineering. This is particularly problematic if marine eco-engineering is being applied as an offset for ecological damage to other habitats or as part of requirements for no net biodiversity loss or even net biodiversity gain from marine construction. The generally stronger effects of complexity at panel than site scales also reinforce the importance of including appropriate control and reference sites in the monitoring and evaluation of eco-engineering interventions [24].

### (c) Implications for management

As sea-levels continue to rise and global energy markets shift to renewable sources such as offshore wind, the number of built structures in our oceans is set to increase, by an anticipated 50–70% by 2030 [12]. Our study has demonstrated that eco-engineering offers a promising approach for enhancing biodiversity at the site scale. However, the benefits of eco-engineering applied through retrofit will depend on the baseline biodiversity on the seawall, and the identity of key environmental stressors, as well as the types of complexity provided. Consequently, where the goal of eco-engineering is to enhance biodiversity, the optimal strategy might be to provide a variety of types of complexity that provide different microhabitats, and mitigate different stressors.

Our study was focused on biomimicry of microhabitats missing from seawalls, but present on rocky shores, the closest natural analogue. Disentangling effects of various aspects of complexity, such as fractal dimension, rugosity and surface area, may however be instructive in designing from first principles microhabitats that maximize benefits to associated species. We expect that those fractal dimensions and rugosities that maximize species diversity in nature may also be most beneficial on artificial substrates.

Additionally, our study tested effects of panel designs at a single study site, and across a relatively narrow tidal elevation gradient. As environmental factors vary not only across tidal elevation but also across estuarine [55] and latitudinal gradients [21], additional tests of panel designs are required across a broad range of environmental conditions. Eco-engineering that is applied without a rigorous scientific evidence base is little more than greenwashing [56].

Our study adds to a growing body of literature that suggests that water-retaining features may be particularly beneficial in bolstering biodiversity on intertidal marine urban structures (e.g. [25,57]). Consequently, we suggest that these may be a particular focus of future eco-engineering interventions in the intertidal space.

## Data Availability

Data for panel- and site-scale sessile and mobile communities are available from the Dryad Digital Repository at https://doi.org/10.5061/dryad.0zpc866zv [58].

## References

[1] Huston M. 1979 A general hypothesis of species diversity. Am. Nat. **113**, 81-101. (10.1086/283366)

[2] Pianka ER. 2000 Evolutionary ecology, 6th edn. San Francisco, CA: Benjamin Cummings.

[3] Kovalenko KE, Thomaz SM, Warfe DM. 2012 Habitat complexity: approaches and future directions. Hydrobiologia **685**, 1-17. (10.1007/s10750-011-0974-z)

[4] Ritchie ME, Olff H. 1999 Spatial scaling laws yield a synthetic theory of biodiversity. Nature **400**, 557-560. (10.1038/23010) 10448857

[5] Hutchinson GE. 1957 Population studies. Cold Spring Harb. Symp. Quant. Biol. **22**, 415-427.

[6] Strain EM et al. 2018 Eco-engineering urban infrastructure for marine and coastal biodiversity: which interventions have the greatest ecological benefit? J. Appl. Ecol. **55**, 426-441. (10.1111/1365-2664.12961)

[7] Strain EMA, Morris RL, Coleman RA, Figueira WF, Steinberg PD, Johnston EL, Bishop MJ. 2018 Increasing microhabitat complexity on seawalls can reduce fish predation on native oysters. Ecol. Eng. **120**, 637-644. (10.1016/j.ecoleng.2017.05.030)

[8] Strain EMA, Cumbo VR, Morris RL, Steinberg PD, Bishop MJ. 2020 Interacting effects of habitat structure and seeding with oysters on the intertidal biodiversity of seawalls. PLoS ONE **15**, e0230807. (10.1371/journal.pone.0230807) 32673342 PMC7365354

[9] Western D. 2001 Human-modified ecosystems and future evolution. Proc. Natl Acad. Sci. USA **98**, 5458-5465. (10.1073/pnas.101093598) 11344294 PMC33234

[10] Hobbs RJ, Higgs ES, Hall C. 2013 Novel ecosystems: intervening in the new ecological world order. Oxford, UK: John Wiley & Sons.

[11] Lai S, Loke LH, Hilton MJ, Bouma TJ, Todd PA. 2015 The effects of urbanisation on coastal habitats and the potential for ecological engineering: a Singapore case study. Ocean Coast. Manag. **103**, 78-85. (10.1016/j.ocecoaman.2014.11.006)

[12] Bugnot AB et al. 2021 Current and projected global extent of marine built structures. Nat. Sustain. **4**, 33-41. (10.1038/s41893-020-00595-1)

[13] Dafforn KA, Glasby TM, Airoldi L, Rivero NK, Mayer-Pinto M, Johnston EL. 2015 Marine urbanization: an ecological framework for designing multifunctional artificial structures. Front. Ecol. Environ. **13**, 82-90. (10.1890/140050)

[14] Gittman RK, Fodrie FJ, Popowich AM, Keller DA, Bruno JF, Currin CA, Peterson CH, Piehler MF. 2015 Engineering away our natural defenses: an analysis of shoreline hardening in the US. Front. Ecol. Environ. **13**, 301-307. (10.1890/150065)

[15] Bulleri F, Chapman MG. 2010 The introduction of coastal infrastructure as a driver of change in marine environments. J. Appl. Ecol. **47**, 26-35. (10.1111/j.1365-2664.2009.01751.x)

[16] Lawrence PJ et al. 2021 Artificial shorelines lack natural structural complexity across scales. Proc. R. Soc. B **288**, 20210329. (10.1098/rspb.2021.0329) PMC8131119 34004129

[17] Munsch SH, Cordell JR, Toft JD. 2017 Effects of shoreline armouring and overwater structures on coastal and estuarine fish: opportunities for habitat improvement. J. Appl. Ecol. **54**, 1373-1384. (10.1111/1365-2664.12906)

[18] Mayer-Pinto M, Cole VJ, Johnston EL, Bugnot A, Hurst H, Airoldi L, Glasby TM, Dafforn KA. 2018a Functional and structural responses to marine urbanisation. Environ. Res. Lett. **13**, 014009. (10.1088/1748-9326/aa98a5)

[19] Mayer-Pinto M, Dafforn KA, Bugnot AB, Glasby TM, Johnston EL. 2018 Artificial structures alter kelp functioning across an urbanised estuary. Mar. Environ. Res. **139**, 136-143. (10.1016/j.marenvres.2018.05.004) 29778444

[20] Firth LB et al. 2014 Between a rock and a hard place: environmental and engineering considerations when designing coastal defence structures. Coastal Eng. **87**, 122-135. (10.1016/j.coastaleng.2013.10.015)

[21] Strain EM et al. 2021 A global analysis of complexity–biodiversity relationships on marine artificial structures. Glob. Ecol. Biogeogr. **30**, 140-153. (10.1111/geb.13202)

[22] Evans AJ, Moore PJ, Firth LB, Smith RK, Sutherland WJ. 2021 Enhancing the biodiversity of marine artificial structures: global evidence for the effects of interventions. Cambridge, UK: Cambridge University Press.

[23] Vozzo ML, Mayer-Pinto M, Bishop MJ, Cumbo VR, Bugnot AB, Dafforn KA, Johnston EL, Steinberg PD, Strain EMA. 2021 Making seawalls multifunctional: the positive effects of seeded bivalves and habitat structure on species diversity and filtration rates. Mar. Environ. Res. **165**, 105243. (10.1016/j.marenvres.2020.105243) 33476978

[24] Chapman MG, Underwood AJ, Browne MA. 2018 An assessment of the current usage of ecological engineering and reconciliation ecology in managing alterations to habitats in urban estuaries. Ecol. Eng. **120**, 560-573. (10.1016/j.ecoleng.2017.06.050)

[25] Evans AJ, Firth LB, Hawkins SJ, Morris ES, Goudge H, Moore PJ. 2015 Drill-cored rock pools: an effective method of ecological enhancement on artificial structures. Mar. Freshw. Res. **67**, 123-130. (10.1071/MF14244)

[26] MacArthur M, Naylor LA, Hansom JD, Burrows MT, Loke LH, Boyd I. 2019 Maximising the ecological value of hard coastal structures using textured formliners. Ecol. Eng. X **1**, 100002. (10.1016/j.ecoena.2019.100002)

[27] Cordell JR, Toft JD, Munsch SH, Goff M. 2017 Benches, beaches, and bumps: how habitat monitoring and experimental science can inform urban seawall design. In Living shorelines (eds DM Bilkovic, MM Mitchell, MK La Peyre, JD Toft), pp. 421-438. Boca Raton, FL: CRC Press. (10.1201/9781315151465-25)

[28] O'Shaughnessy KA et al. 2021 Spatially variable effects of artificially created physical complexity on subtidal benthos. Front. Ecol. Evol. **9**, 690413. (10.3389/fevo.2021.690413)

[29] Malumbres-Olarte J, Vink CJ, Ross JG, Cruickshank RH, Paterson AM. 2013 The role of habitat complexity on spider communities in native alpine grasslands of New Zealand. Insect Conserv. Divers. **6**, 124-134. (10.1111/j.1752-4598.2012.00195.x)

[30] Loke LH, Todd PA. 2016 Structural complexity and component type increase intertidal biodiversity independently of area. Ecology **97**, 383-393. (10.1890/15-0257.1) 27145613

[31] Connell JH. 1961 The influence of interspecific competition and other factors on the distribution of the barnacle *Chthamalus stellatus*. Ecology **42**, 710-723. (10.2307/1933500)

[32] Dayton PK. 1971 Competition, disturbance, and community organization: the provision and subsequent utilization of space in a rocky intertidal community. Ecol. Monogr. **41**, 351-389. (10.2307/1948498)

[33] Underwood AJ, Jernakoff P. 1981 Effects of interactions between algae and grazing gastropods on the structure of a low-shore intertidal algal community. Oecologia **48**, 221-233. (10.1007/BF00347968) 28309804

[34] Boaventura D, Alexander M, Della Santina P, Smith ND, Ré P, da Fonseca LC, Hawkins SJ. 2002 The effects of grazing on the distribution and composition of low-shore algal communities on the central coast of Portugal and on the southern coast of Britain. J. Exp. Mar. Biol. Ecol. **267**, 185-206. (10.1016/S0022-0981(01)00372-0)

[35] Stillman J, Somero G. 1996 Adaptation to temperature stress and aerial exposure in congeneric species of intertidal porcelain crabs (genus *Petrolisthes*): correlation of physiology, biochemistry and morphology with vertical distribution. J. Exp. Biol. **199**, 1845-1855. (10.1242/jeb.199.8.1845) 9319758

[36] McAfee D, Bishop MJ, Yu TN, Williams GA. 2018 Structural traits dictate abiotic stress amelioration by intertidal oysters. Funct. Ecol. **32**, 2666-2677. (10.1111/1365-2435.13210)

[37] Vermeij GJ. 1987 Evolution and escalation: an ecological history of life. Princeton, NJ: Princeton University Press.

[38] Sih A, Crowley P, McPeek M, Petranka J, Strohmeier K. 1985 Predation, competition, and prey communities: a review of field experiments. Annu. Rev. Ecol. Syst. **16**, 269-311. (10.1146/annurev.es.16.110185.001413)

[39] Anderson MJ, Gorley RN, Clarke KR. 2008 *PERMANOVA+ for PRIMER: guide to software and statistical methods.* Plymouth, UK: PRIMER-E.

[40] Anderson MJ, Robinson J. 2003 Generalized discriminant analysis based on distances. Aust. N. Z. J. Stat. **45**, 301-318. (10.1111/1467-842X.00285)

[41] Connor EF, McCoy ED. 1979 The statistics and biology of the species-area relationship. Am. Nat. **113**, 791-833. (10.1086/283438)

[42] Aguilera MA, Broitman BR, Thiel M. 2014 Spatial variability in community composition on a granite breakwater versus natural rocky shores: lack of microhabitats suppresses intertidal biodiversity. Mar. Pollut. Bull. **87**, 257-268. (10.1016/j.marpolbul.2014.07.046) 25103901

[43] Morris RL, Porter AG, Figueira WF, Coleman RA, Fobert EK, Ferrari R. 2018 Fish-smart seawalls: a decision tool for adaptive management of marine infrastructure. Front. Ecol. Environ. **16**, 278-287. (10.1002/fee.1809)

[44] Krassoi FR, Brown KR, Bishop MJ, Kelaher BP, Summerhayes S. 2008 Condition-specific competition allows coexistence of competitively superior exotic oysters with native oysters. J. Anim. Ecol. **77**, 5-15. (10.1111/j.1365-2656.2007.01316.x) 18177325

[45] Toft JD, Ogston AS, Heerhartz SM, Cordell JR, Flemer EE. 2013 Ecological response and physical stability of habitat enhancements along an urban armored shoreline. Ecol. Eng. **57**, 97-108. (10.1016/j.ecoleng.2013.04.022)

[46] Morris RL, Chapman MG, Firth LB, Coleman RA. 2017 Increasing habitat complexity on seawalls: investigating large- and small-scale effects on fish assemblages. Ecol. Evol. **7**, 9567-9579. (10.1002/ece3.3475) 29187990 PMC5696408

[47] Dafforn KA. 2017 Eco-engineering and management strategies for marine infrastructure to reduce establishment and dispersal of non-indigenous species. Manag. Biol. Invas. **8**, 153-161. (10.3391/mbi.2017.8.2.03)

[48] Moreno-Mateos D et al. 2017 Anthropogenic ecosystem disturbance and the recovery debt. Nat. Commun. **8**, 14163. (10.1038/ncomms14163) 28106039 PMC5263871

[49] Bracewell SA, Clark GF, Johnston EL. 2018 Habitat complexity effects on diversity and abundance differ with latitude: an experimental study over 20 degrees. Ecology **99**, 1964-1974. (10.1002/ecy.2408) 29846936

[50] Chapman MG. 2003 Paucity of mobile species on constructed seawalls: effects of urbanization on biodiversity. Mar. Ecol. Prog. Ser. **264**, 21-29. (10.3354/meps264021)

[51] Moreira J, Chapman MG, Underwood AJ. 2006 Seawalls do not sustain viable populations of limpets. Mar. Ecol. Prog. Ser. **322**, 179-188. (10.3354/meps322179)

[52] Mormul RP, Thomaz SM, Takeda AM, Behrend RD. 2011 Structural complexity and distance from source habitat determine invertebrate abundance and diversity. Biotropica **43**, 738-745. (10.1111/j.1744-7429.2011.00762.x)

[53] Mayer-Pinto M, Matias MG, Coleman RA. 2016 The interplay between habitat structure and chemical contaminants on biotic responses of benthic organisms. PeerJ **4**, e1985. (10.7717/peerj.1985) 27168991 PMC4860324

[54] Esquivel‐Muelbert JR, Lanham B, Martínez‐Baena F, Dafforn KA, Gribben PE, Bishop MJ. 2022 Spatial variation in the biotic and abiotic filters of oyster recruitment: implications for restoration. J. Appl. Ecol. **59**, 953–964. (10.1111/1365-2664.14107)

[55] Clifton GA, Dafforn KA, Bishop MJ. Submitted. The benefits of adding complexity to seawalls vary across environmental gradients. Ecol. Eng.

[56] Firth LB, Airoldi L, Bulleri F, Challinor S, Chee SY, Evans AJ, Hanley ME, Knights AM, O'Shaughnessy K, Thompson RC, Hawkins SJ. 2020. Greening of grey infrastructure should not be used as a Trojan horse to facilitate coastal development. J. Appl. Ecol. **57**, 1762-1768. (10.1111/1365-2664.13683)

[57] Browne MA, Chapman MG. 2014 Mitigating against the loss of species by adding artificial intertidal pools to existing seawalls. Mar. Ecol. Prog. Ser. **497**, 119-129. (10.3354/meps10596)

[58] Bishop MJ, Vozzo ML, Mayer-Pinto M, Dafforn KA. 2022 Data from: Complexity–biodiversity relationships on marine urban structures: reintroducing habitat heterogeneity through eco-engineering. Dryad Digital Repository. (10.5061/dryad.0zpc866zv) PMC9234820 35757880

